# The Treatment of the Morphine Habit in General Practice

**Published:** 1908-10

**Authors:** Geo. H. Lehmann

**Affiliations:** Augusta, Ga.


### THE TREATMENT OF THE MORPHINE HABIT IN GENERAL PRACTICE.*

MORPHINOMANIA—THE MORBID DESIRE FOR MORPHINE—THE OPIUM HABIT.

BY GEO. H. LEHMANN, AUGUSTA, GA.

Before taking up this subject, I wish to make it plain to you gentlemen that I consider the confinement in some well regulated institution of all morphine fiends absolutely essential, and where the patient can stand the expense, this should always be done. But, unfortunately, we find a great many of these poor creatures who are unable to bear the expense of an institution with its skilled physicians and attendants. To this class the physician is called upon from time to time to treat. Now, the averge practitioner has very little sympathy for this class of unfortunates, for experience has taught him that no reliance can be placed in this class of patients, while the treatment instituted is positively worthless or unsatisfactory. The morphine habit is often acquired by patients for whom the drug has been prescribed by a physician to control obstinate pain or sleeplessness, or from self-administration, or certain patent medicines. It is more frequent in women than in men. Physicians are often addicted to this pernicious habit, in fact, more so than any other professional body. Morphine is taken either by the mouth or hypodermatically, and while some few subjects continue to take the same small quantity of the drug, the tendency is to gradually increase the dose until 40 grains or more are employed.

In the East, opium eating and smoking are very common, yet the Orientals seem to stand it without any marked bad effect. The continued use of small doses of morphine may for a long time result in no marked manifestations other than a craving for the drug, but sooner or later the functions of both body and mind become affected. While under the influence of the morphine the patient may feel well, but as the effects disappear, mental disquietude, depression, nausea and abdominal pains follow, which can only be relieved by further recourse to the drug. The character of the morphinomaniac becomes deteriorated and

*Read before the Richmond County Medical Society, Augusta, Ga., July 14, 1908.

is typified by lack of self control and of moral sense—the patient loses all sense of right and wrong. He will lie and steal in the most degrading way, especially if his desire is to obtain the drug, and absolutely no statement that he makes can be trusted. He neglects his work and lets his business go to ruin. He becomes anaemic, suffers from loss of appetite, indigestion, dry mouth, sluggish bowels, and a foul tongue. The nails are brittle, the skin is dry, the hair turns gray early and falls out. There is sexual impotence in the male, no erections take place, no semen is secreted. In the female there is amenorrhea, and the flow of milk is stopped, but there is polyuria. The pupils are small, there is loss of muscular power, slight ataxia and tremor in severe cases. The arms, legs, and in fact, every portion of the body that can be reached, are scarred by marks of the needle. Certain subjects live to moderate old age, and even, though the habit continues, are able to transact the usual duties of life. These, however, are the rare individuals who get along on a small and not increased quantity of the drug.

A common feature of all narcotic inebriety is the frequent perversion of the affections. Love is transformed into hatred, and the narcomaniac not unseldom loathes the sight of the devoted companion whom, in his prenarcotic years, he cherished with the tenderest affection. Opium transforms the manly, hightoned, pleasant companion into an effeminate, driveling, querulous bore. In some localities, especially in China, the opium degradation is so terrible that gross immorality abounds. So intense is the crave that a man has been known to mortgage his mother and sell his wife to gratify it. One man sold his wife for 12 pounds and smoked the proceeds. The crave robs a man of his resources, unfits him for work, and hurries him to an untimely end. J. L. Maxwell, in the Lancet of January 2nd, 1893, says that one hundred thousand persons commit suicide by opium every year in China. In Hardai, of 180 suicides in three years, 97 were from opium ,and 80 per cent of these being females. Morphine produces abulic states which predispose to impreative conceptions, leading to theft usually of senseless nature.

Diagnosis.—Opiomaniacs and Morphinomaniacs are often difficult of detection, especially is this true if they have a supply of the drug about them. Norman Kerr says: In one case a brilliant young medicine student had habitually taken opium for two years without the habit having been suspected by the chum who shared his room. The truth was disclosed unexpectedly, owing to an unusually large dose having been taken by mistake. It is astonishing how dextrous with the hypodermic syringe the inebriate becomes. The diagnosis of morphinism is easy. On examining the arms, scars caused by the use of the hypodermic syringe, are readily seen. To find morphine in the urine, take twenty ounces of the suspected urine, and if not acid in reaction, make it so by adding diluted hydrochloric acid and concentrate to about three ounces, when it is allowed to stand in a cool place for twelve hours; then filtered. To the filtrate is added sufficient sodium carbonate to make it alkaline. It is then allowed to stand twelve hours, filtered, and the precipitate collected and washed with distilled water made slightly alkaline with sodium carbonate and dried. The dried precipitate is digested with pure alcohol at a gentle heat and filtered. This is evaporated to dryness. The residue is disolved with diluted sulphuric acid and tested for morphine by iodic acid test. Iodic acid is reduced by morphine rendering color of solution brownish from free iodine, changing to bluish black on addition of starch water.

ETIOLOGY.—The Medical Record in an editorial of June, 1897, says that of male morphinist, the medical profession supplies the largest number—40 per cent. Men of leisure comes next with 15 per cent. Then merchants with 8 per cent., while clergymen, peasants and politicians occupy the lowest position on the list. Women of means are the most numerous class among the females, 43 per cent., followed by the wives of medical men with 10 per cent. A sedative for the relief of pain has been the origin with many. Neuralgic pains being generally relieved as if by magic when introduced subcutaneously. J. B. Mattison, in the Medical Record of January, 1895, says that neuralgia is the most prolific cause of morphinism. Injudicious medical prescriptions has had much to answer for in introducing the practice of the auto-injection of narcotics.

PATHOLOGY.—The pathological changes observed in morphinism are few and limited. The shrunken and withered appearance of the habitual inebriate is a fair representation of his internal condition. Erlenmeyer, in the American Journal of Medicine of April, 1904, says that morphine in the body, by taking up oxygen, is changed into oxidimorphine, which later gives rise to symptoms of abstinence. Organic lesions are comparatively rare. Opium may be a contributary cause of paralysis, but it does not act directly as a paralyzer.

TREATMENT.—In hospital practice, opium or any of its derivatives, may be withdrawn suddenly, and hyoscine hydrobromate substituted, but this method, in my opinion, is positively cruel, causing untold suffering on the part of the patient from three to four days. J. C. Verco, in the Australian Medical Gazette of March 20, 1899, says that the following method of treating chronic morphinism is simple, safe and satisfactory. A mixture containing five grains of bromide of soda to a drachm is ordered, and a drachm of the mixture is given every three hours; the dose is increased by a drachm every day. By the time the patient is taking 1-2 drachm doses of the bromide every three hours for a day or two, he will probably be able to do without his morphine. If the drowsiness by this time is profound, the bromide may be withdrawn; if not very deep the bromide may be gradually withdrawn. On May 22, 1901, C. B. A. Barber came to my office and informed me that he wished to place his case in my hands for treatment. This man was a confirmed morphine fiend. Some twelve or thirteen years before he stated that he suffered from rheumatism, and his physician gave him morphine hypodermatically, sometimes as often as three or four times daily for relief. The physician finally realized that he was about to make a fiend of his patient and ceased the administration of the drug. He saw nothing of the man for several days, and when he did, inquired why he had not called at his office for further treatment. He stated to the physician that when we refused to give him the morphine, he immediately went to a near-by drug store and bought a hypodermic syringe and morphine and began treating himself. He kept this treatment up persistently for about thirteen years, when he came to me for treatment. At the time he was taking one drachm of the powdered drug daily. His arms, legs and body were a mass of scars—I never before saw a more pitiful specimen of humanity. He was anxious to be cured of the habit, and I told him I would take his case if he would pledge me his hearty co-operation in the treatment. This he readily consented to do. I relieved him of his syringe and told him that whenever he felt that he must have the drug, come to my office and I would administer the necessary quantity. For the first three days I gave him six grs. four times daily, and at the end of the week I had reduced the quantity to fifteen grs. given in three doses. Keith's Concentrated Tincture Avena Sativa was given from the first in increasing doses, beginning with ten gtts. Saturated solution sodium iodide was given before meals in milk in increasing doses, for the purpose of bringing about a rapid elimination of the drug. Strych. sulph. was given with the morphine hypodermatically beginning with 1-60 grs. and gradually increased as the morphine was decreased. At the end of five weeks I had completely withdrawn the drug and my patient had gained about ten pounds. He was dismissed with a prescription for strychnia granules grs. 1-40, to be taken every four hours.

This man lived in Augusta for nearly three years afterwards and during that time there was no relapse, subsequently I was informed that he became addicted to strong drink, and finally lost his position and left Augusta, and of course, passed from observation.

Case No. 2.—Mrs. W., a distant relative, residing in western Georgia, had been addicted to the drug for five years. Her physician gave it to her for the relief of neuralgia from which she was a chronic sufferer. She purchased a syringe and afterwards administered the drug herself. Her husband placed her in the Woolleys Institute of Atlanta, and the drug was suddenly withdrawn and hyoscine hydrobromate substituted in large doses. She remained in a semi-conscious state for about seventy-two hours. After recovering from the effects of the hyoscine, she passed into a state of nervous collapse, which so frightened the physicians that they immediately summoned her husband to Atlanta. Three days later she left the institute, saying she could not take the treatment. Three months afterwards she came to Augusta and remained at my house during the course of treatment. She was taking about six grs. daily hypodermatically. The treatment given was the same as given in the first case. In three weeks the drug was completely withdrawn and she had gained six pounds. I saw this patient about two months ago. She had gained about thirty-five pounds since I dismissed her four years ago. She stated that during that time she had several severe attacks of neuralgia, but would never permit her physician to give her morphine—she had a perfect horror of the drug, and under no condition would she take it again.

Case No. 3.—L. A.—A denizen, of the Red Light District, in 1904 applied to me for treatment. I refused to take her case unless she would consent to go to the hospital, which she did. At the time she was taking about 10 grs. of the drug daily, hypodermatically. On reaching the hospital she was placed in bed, where she remained for two weeks when the drug was completely withdrawn. In this case nothing was used except the solution of iodide and the avena sativa, plenty of nourishment, rich milk and broths. She was discharged at the end of three weeks. In this case there was a relapse, which was due to her physician giving her morphine to control the pain of facial erysipelas. That a relapse would come about sooner or later in this patient was to be expected, as her very life, surroundings and environments were all predisposing factors. I have a record of some twenty-two or three cases, and the cases just mentioned were taken from my records just as they came in one, two, three order. I believe that 80 per cent. of the morphine fiends can be cured., 40 per cent. of these will relapse from time to time, while fully 40 per cent. remain permanently cured.

RECAPITULATION.—Much may be done by the physician in the way of prevention of the morphine habit. The indiscriminate prescribing of the drug can not be too strongly condemned, and it is a positive crime to put a hypodermic syringe into the hands of a patient to be used in the control of pain or sleeplessness. Be frank with your patient—make it plain that you can and will cure him with his co-operation, when possible, place him in bed for a few days, and by all means insist on his remaining in doors during the course of treatment. Begin with 15 gtts. of Keith's concentrated tincture avena sativa four times daily in hot water, gradually increasing the dose until thirty or forty gtts. are given. A fullness at the base of the brain will indicate that the dose must not be increased, and a pain in that region suggests that an overdose has been given. Avena sativa is a *nerve stimulant, tonic, laxative, solvent* and *diuretic*. The morphine is gradually reduced, while strychnine sulph. given with the morphine, is gradually increased, and, as a rule, the drug can be completely withdrawn in from ten to fourteen days. Sat. solution sodium iodide is given in increasing doses before meals in milk, beginning with ten gtts. This brings about the rapid elimination of the drug. In some cases trional in fifteen grs. doses at bed time will have to be given. In women of highly nervous temperament, I have found that John B. Daniels concentrated tincture of passiflora incarnata has a wonderful sedative effect upon the nervous system. So effective is this drug that I rely upon it entirely now, whereas before I used the bromides. Hardly any two cases can be treated exactly alike, therefore the medical profession should alter the treatment in such cases where the above is not applicable, using their judgment, though following along the same general lines.

In conclusion, I will say be patient, be merciful to these poor unfortunates, for they are not entirely to blame for their wretched condition.

